# Supplementary material for: Learning curve and surgical outcome of robotic assisted colorectal surgery with ERAS program
Source: Sci Rep. 2022 Nov 29;12:20566. doi: 10.1038/s41598-022-24665-w (PMC9709162; doi:10.1038/s41598-022-24665-w)
Supplement: Supplementary file 1 — Supplementary Information 1. [file 41598_2022_24665_MOESM1_ESM.zip › LSC Raw data-Ñ[▒K/049/HS_ñw╝╨░O▒Kñσ.pdf]

正式摘要(電子簽章：0000001787923848)

W52 / W52 ☐ 英文標題(注意:無英文姓名-請補病人基本檔!) ☐【讀取】憑證

臺中榮民總醫院

出院病歷摘要

[redacted]

| | |
|---|---|
| (8)流水編號 | |
| (9)入院日期 | 108/10/07 (2019) 直腸外科 W52 - 162 |
| (10)轉科日期 | |
| (11)出院日期 | 108/10/30 (2019) 住院天數計 23 日 |

## 診斷

- 入院 :1. Colon cancer, adenocarcinoma, cTxN0M0, Dukes A-B
  2. Right breast cancer, IDC, [cT3N1M0,ER- PR- HER2(3+) Ki67(70%)], with pleural metastases and lymphagitis, s/p neoadjuvant chemotherapy, hormone therapy, target therapy, and adjuvant radiotherapy since 2015, s/p right total mastectomy +ALND on 2015/08/28
  3. Febrile neutropenia, s/p tazocin 2019/09/16-09/23
  4. HCV carrier under GI OPD f/u, s/p Sofosbuvir/Daclatasvir since 2016/09/13, totally 12 weeks.(2016/12/12 Target Not Detected )
  5. Liver hemangioma, by 2016/8/8 sonography
- 出院 :1. A-colon cancer, adenocarcinoma, pT3N0M0, stage 2A. S/p right hemicolectomy and anastomosis on 10/8
  2. Intra-abdominal abscess formation, Abx with Cefazolin+Metronidazol 10/17, Flomoxef 10/17-10/21, Brosym 10/21-10/30
  3. Right breast cancer, IDC, [cT3N1M0,ER- PR- HER2(3+) Ki67(70%)], with pleural metastases and lymphagitis, s/p neoadjuvant chemotherapy, hormone therapy, target therapy, and adjuvant radiotherapy since 2015, s/p right total mastectomy +ALND on 2015/08/28
  4. Febrile neutropenia, s/p tazocin 2019/09/16-09/23
  5. HCV carrier under GI OPD f/u, s/p Sofosbuvir/Daclatasvir since 2016/09/13, totally 12 weeks.(2016/12/12 Target Not Detected )
  6. Liver hemangioma, by 2016/8/8 sonography

## 主訴

- Intermittent bloody stool was found during last admission and colonoscopy biopsy showed adenocarcinoma.

## 病史

- This 47 y/o female had the history of
  1. Colon cancer, adenocarcinoma, cTxN0M0, Dukes A-B
  2. Right breast cancer, IDC, [cT3N1M0,ER- PR- HER2(3+) Ki67(70%)], with pleural metastases and lymphagitis, s/p neoadjuvant chemotherapy, hormone therapy, target therapy, and adjuvant radiotherapy since 2015, s/p right total mastectomy +ALND on 2015/08/28
  3. Febrile neutropenia, s/p tazocin 2019/09/16-09/23
  4. HCV carrier under GI OPD f/u, s/p Sofosbuvir/Daclatasvir since 2016/09/13, totally 12 weeks.(2016/12/12 Target Not Detected )
  5. Liver hemangioma, by 2016/8/8 sonography

  Intermittent bloody stool was noted during her last admission for the neutropenic fever. Upper GI and lower survey was done, and colonoscopy found ileocecal valve tumor, biopsy was done, and the pathology report showed Adenocarcinoma. Abdominal CT was arranged and C/W colon cancer over cecum and proximal A-colon, no other specific findings noted at liver. Surgical intervention was suggested, and the patient agreed.
  She was admitted for pre-OP assessment and further management.

## 體檢發現

- chest wall scar:prominant erythematous change

  Chest CT on 1060612:SD
  Chest CT on 1061102:SD
  Chest CT on 1070302:SD
  chest CT on 1070626:SD
  chest CT on 1071024:PD
  chest CT on 10800214:SD

  1070823 No side effect
  1071004 No side effect
  1071108 No side effect
  1071107 HGB: 11.1 (Grade1)
  1071220 No side effect
  1080131 No side effect
  1080314 No side effect
  1080313 HGB: 7.3 (Grade3)
  1080422 No side effect
  1080603 No side effect
  1080729 No side effect

  Response Evaluation:Stable Disease,
  (Chemotherapy):Gemzar inj 200mg,
  (Targeted therapy):(IV)Herceptin inj 440mg,Perjeta inj 420mg,
  1080930 No side effect
  - General appearance :
    - easy looking , conscious clear , without cardiopulmonary distress
    - Vital signs自動帶入date 體溫(BT) 脈搏(P) 呼吸(R) 血壓(BP) 身高(BH) 體重(BW) BMI
      20191007 36.6℃ 113次/分 18次/分 112/69mmHg 161.5cm 54.9kg 21.0
    - Vital signs: B.P. 112/69 mmHg , P.R. 113 /min , R.R. 18 /min , B.T. 36.6 ℃
  - HEENT :
    - Eyes: no anemic conjunctiva, no icteric sclera, normal EOM
    - ENT: no otorrhea, no rhinorrhea, no erythematous mucosa, no injected tonsil, no deformity
  - Neck: supple, freely movable, trachea midline,no deformity, no engorged jugular vein , no palpable thyroid or lymph node enlargement
  - Chest:
    - Inspection: Grossly normal, symmetric and fully expansion, no accessory muscle used, no operation scar, no spider angiomata, no gynecomastia
    - Palpation: no palpable mass or nodule, no subcutaneous emphysema, no tenderness
    - Ausculation: clear breathing sound
  - Heart:
    - Palpation: no thrill, no heave
    - Percussion: normal heart size
    - Ausculation: regular heart beat, normal S1, S2, no gallop, no murmur
  - Extremities: No deformity, full ranged of motion without limitation, no clubbing finger or nail cyanosis, no pitting edema, no ulcer or pigmentation, no obvious skin lesion, no varicose vein, no bone, joint or muscle tenderness

  **CRS CONDITION**
  - Karnofsky performance status: (100) Normal, no complaints, no evidence of disease
  - PE of Abdomen:
  - __1. Inspection: flat
  - __2. Ausculation: normal active bowel sound
  - __3. Palapation: soft, no tenderness
  - __4. Percussion: -
  - CEA: 20.21 ng/ml
  - Imaging Studies:

- Colonoscope report: DIAGNOSIS: 1. Ileocecal valve tumor R/O CRC R/O Metastasis s/p biopsy 2. Internal hemorrhoids, mild
- Sono , CT of abdomen and chest Films:檢驗項目 :CT ABDOMEN-SURVEY 報告日期 :1080924

  主 訴 :
  Result:
  Finding:
  Imaging Report Form for Colorectal Carcinoma (AJCC 8th)
  1. Imaging Date: 20190924
  Imaging Modality, Imaging by
  CT scan
  Imaging Protocol:
  Post-contrast CT of abdomen with oral and IV contrast enhancement.
  2. Tumor location / Size
  Location:
  Cecum
  Ascending
  Size:
  Non-measurable
  3. Tumor invasion
  Tumor invades non-peritonealized pericolonic or perirectal tissues
  4. Regional nodal metastasis
  No
  5. Distant metastasis (In this study)
  No or Equivocal
  6. Other findings:
  > Irregular wall thickening at the cecum and proximal A-colon(Axi#52-61), with periphery stranding appearance. C/W colon cancer.
  > Several smaller than 1.0cm mesentery LNs over RLQ of abdomen.
  > Several smaller than 1.0cm over retroperitoneum.
  > No other specific findings noted at liver, GB, pancreas, spleen, bil. kidneys and bil. adrenal glands.
  > History of right breast cancer S/P MRM.
  > Persistent bil. pleural effusion, more on right side.
  > R/O radiation pneumonitis of lungs, the visible lungs are without obvious change as compared with previous Chest CT on 20190624.
  > No obvious lesion noted in visible bony structure.
  Impression:
  C/W colon cancer over cecum and proximal A-colon.
- Figures:
  1. colon & rectum

## 手術日期及方法

- 手術日期及方法20191008 Laparoscopic right colectomy and anastomosis

## 住院治療經過

- After admission, pre-OP assessment was done, and the lab data showed no significant abnormality needed to be corrected before the surgery. Laparoscopic right colectomy and anastomosis was performed on 2019/10/8 smoothly. After the surgery, she was sent back to general ward for post OP care. We let her try water on 10/13, and no discomfort was complained. Mild abdominal distension was noted on 10/14, and KUB showed moderate bowel gas. We let her try liquid diet on 10/15, semi-liquid on 10/16. Drainage tube was removed on 10/16. Abdominal pain with mild fever was noted on 10/17. Abdominal CT was arranged, and revealed no intra-abdominal free air, inflammation change around anastomosis, and susp. abscess formation at right subhepatic area near anastomotic site. We gave her empiric antibiotics with Flomoxef on 10/17, and let her NPO. The fever subside after antibiotics treatment. The patient complained right flank pain on 10/21, so we arranged abdominal CT again to r/o abscess progression, and the CT showed decreased abscess size. Wound culture was obtained, and the report showed E. coli and Pseudomonas. Therefore, antibiotics was shifted to Brosym on 10/21. CVC was placed on 10/21 due to no accessible peripheral line. Her condition was stablized. No fever and abdominal pain was complained. The follow-up lab data showed no leukocytosis. We kept Brosym from 10/21-10/30. CVC was removed on 2019/10/30. Under stable condition, she was discharged on 2019/10/30 with oral antibiotics. OPD follow-up was arranged.

## 合併症

- Intra-abdominal abscess formation, Abx with Cefazolin+Metronidazol 10/17, Flomoxef 10/17-10/21, Brosym 10/21-10/30

## 一般檢查

- 累積報告 :

| DATE | TIME | ALB | TP | BIL.T | BIL.D | ALKP | AST | ALT | LDH | CHOL | TG | HDL-C | LDL | UA | NH3 | AMY |
|---|---|---|---|---|---|---|---|---|---|---|---|---|---|---|---|---|
| 1080916 | 1339 | | | 0.3 | | 100 | 35 | | | | | | | | | |
| 1080920 | 0858 | | | 0.68 | | 107 | | 25 | 221 | | | | | | | |
| 1080923 | 0843 | | | 0.36 | | 139 | 23 | 20 | 235 | | | | | | | |
| 1080930 | 1307 | 4.1 | | 0.30 | 0.09 | 118 | 28 | 19 | 207 | | | | | | | |
| 1081007 | 1656 | 4.2 | | 0.4 | 0.1 | | 27 | 15 | | | | | | | | |
| 1081007 | 1656 | 4.2 | | | | | | | | | | | | | | |
| 1081008 | 1531 | 3.5 | | 0.3 | 0.1 | | 25 | 16 | | | | | | | | |
| 1081014 | 0917 | 3.3 | | 0.41 | | | | 9 | | | | | | | | |
| 1081017 | 1447 | 3.7 | | 0.5 | | | | 7 | | | | | | | | |
| 1081018 | 1416 | | | | | | | | | 135 | 95 | | | | | |
| 1081021 | 0935 | 3.4 | | 0.28 | | | | 9 | | | | | | | | |
| 1081024 | 0848 | 3.2 | | 0.15 | | | | 12 | | | | | | | | |
| 1081028 | 0921 | 3.8 | | 0.35 | | | | 22 | | | | | | | | |

| DATE | TIME | WBC | RBC | HGB | HCT | MCV | PLT | BLST | NEUT | BAND | SEG | LYM | MON | EOS | BAS |
|---|---|---|---|---|---|---|---|---|---|---|---|---|---|---|---|
| 1080916 | 1337 | 1740 | | 8.2 | | | 83 | | | | 72 | 24 | 4 | | |
| 1080917 | 0557 | 2570 | 2.11 | 6.6 | 20.0 | 94.8 | 67 | | 79.4 | | | 14.8 | 5.4 | 0.4 | 0.0 |
| 1080918 | 0828 | 3770 | 2.61 | 7.6 | 23.9 | 91.6 | 64 | | 70.5 | | | 22.0 | 6.9 | 0.3 | 0.3 |
| 1080920 | 0842 | 1480 | 4.10 | 12.0 | 37.0 | 90.2 | 90 | | | | 44.0 | 30.0 | 24.0 | 2.0 | 0.0 |
| 1080923 | 0852 | 14380 | 3.98 | 11.8 | 36.7 | 92.2 | 135 | | 85.5 | | | 9.8 | 4.5 | 0.1 | 0.1 |
| 1080926 | 0828 | 4210 | 4.16 | 12.2 | 38.2 | 91.8 | 188 | | 65.8 | | | 23.3 | 10.2 | 0.2 | 0.5 |
| 1080930 | 1307 | 5960 | 3.92 | 11.7 | 36.7 | 93.6 | 292 | | 79.5 | | | 14.6 | 5.2 | 0.2 | 0.5 |
| 1081007 | 1652 | 5360 | 3.63 | 10.9 | 34.5 | 95.0 | 318 | | 75.9 | | | 15.5 | 8.4 | 0.0 | 0.2 |
| 1081008 | 1531 | 4070 | 3.24 | 9.7 | 30.3 | | 247 | | 90.2 | | | 7.9 | 1.7 | 0.0 | 0.2 |
| 1081014 | 0909 | 5180 | 3.27 | 9.7 | | | 281 | | 81.8 | | | 9.8 | 6.8 | 1.2 | 0.4 |
| 1081017 | 1215 | 14250 | 4.03 | 12.2 | | | 322 | | 90.8 | | | 4.8 | 4.1 | 0.1 | 0.2 |
| 1081021 | 1238 | 6850 | 3.20 | 9.4 | | | 295 | | 85.2 | | | 8.9 | 4.4 | 1.2 | 0.3 |
| 1081024 | 0859 | 4320 | 2.87 | 8.4 | | | 236 | | 76.6 | | | 14.4 | 5.3 | 3.2 | 0.5 |
| 1081028 | 0957 | 4770 | 3.84 | 11.1 | | | | | 78.4 | | | 13.4 | 5.5 | 2.1 | 0.6 |

| DATE | TIME | NA | K | CL | CA | BUN | CREAT | Mg | P | CRP | ESR | PCT | LIPASE | LACTATE |
|---|---|---|---|---|---|---|---|---|---|---|---|---|---|---|
| 1080916 | 1339 | 137 | 3.5 | | | 11 | 0.59 | | | 2.701 | | | | 10.0 |
| 1080920 | 0858 | 140 | 3.7 | | | 7 | 0.64 | | | | | | | |
| 1080923 | 0843 | 141 | 3.5 | | 8.9 | 9 | 0.62 | | | | | | | |
| 1080930 | 1307 | 140 | 4.1 | | 9.5 | 12 | 0.55 | | | | | | | |
| 1081007 | 1656 | 137 | 4.2 | 102 | 9.0 | 16 | 0.64 | | | | | | | |
| 1081008 | 1531 | 139 | 4.1 | | | 9 | 0.51 | | | | | | | |
| 1081014 | 0917 | 139 | 4.5 | | 8.8 | 10 | 0.53 | | | | | | | |
| 1081017 | 1447 | 138 | 3.8 | | 9.0 | 11 | 0.47 | | | | | | | |
| 1081018 | 1416 | | | | | | | 1.8 | 3.1 | | | | | |
| 1081021 | 0935 | 142 | 4.0 | | 8.7 | 8 | 0.48 | | | | | | | |
| 1081024 | 0848 | 142 | 3.8 | | 8.5 | 8 | 0.45 | | | | | | | |
| 1081028 | 0921 | 140 | 4.3 | | 9.1 | 9 | 0.51 | | | | | | | |

| DATE | TIME | GLUCOSE | KETONE | CK | CKMB | PT-P | PT-C | APTT-P | APTT-C | TRO-T |
|---|---|---|---|---|---|---|---|---|---|---|
| 1080916 | 1339 | 111 | | | | | | | | |
| 1080930 | 1307 | 104 | | | | 10.0 | 10.1 | 27.0 | | |

| DATE | TIME | SP-GR | PH | PROT | GLUC | KETON | BILI | UROB | OB | NITR | LEU | RBC | WBC | EPCEL |
|---|---|---|---|---|---|---|---|---|---|---|---|---|---|---|
| 1080916 | 1300 | 1.030 | 6.0 | Trace | Negative | Negative | Negative | 0.2 | Negative | Negative | Negative | 0-2 | 0-2 | 0-1 |
| 1081014 | 1418 | 1.012 | 6.0 | Negative | Negative | 3+ | Negative | 0.2 | 3+ | Negative | Trace | + | 0-2 | 0-1 |

| DATE | TIME | STOOL-OB | FAT | RBC | WBC | PARA |
|---|---|---|---|---|---|---|
| 1080918 | 0938 | 4+ | | 2+ | 0 - 2 | Not Found |

| | | | | | | |
|---|---|---|---|---|---|---|
| 1080923 | 1655 | | | - | - | Not Found |

- 放射線報告 :檢驗項目 **:CT ABDOMEN-SURVEY** 報告日期 **:1081022**

主　訴 :
Result:
Finding:
Pre and Post-contrast CT of abdomen with oral and IV contrast enhancement.
> Adenocarcinoma of ascending colon s/p right hemicolectomy
> Decreased amount of ascites
* Focal loculated fluid accumulation with wall enhacement over liver surface to anatomosis site and body surface, abscess decreased size as compared with previous study on 20191017.
> No other specific findings noted at liver, GB, pancreas, spleen, bil. kidneys and bil. adrenal glands.
> Persistent bil. pleural effusion, more on right side.
> A consolidative lesion with calcification over right lower lung, stable compared with previous study on 20190624
> Fibrotic change over left lower lung
> No obvious lesion noted in visible bony structure.
Impression:
As above description

## 特殊檢查

## 病理報告

- 活體組織切片病理報告　手術日期：**108/10/08**　病理號：**S0835978**

診斷醫師:李欣悅 報告日期:108/10/16
Pathologic diagnosis:
Ascending colon, terminal ileum, and appendix, laparoscopic right hemicolectomy --- Adenocarcinoma, moderately differentiated, of ascending colon. Tumor invaded to mesocolic soft tissue with ileocecal valve involvement. The appendix, bilateral cut ends and mesocolic lymph nodes (0/24) are free of tumor.
Ancillary study for diagnosis:
IHC stains for MLH1, MSH2, PMS2 and MSH6 are done on section F. All above markers are preserved.
Prognostic and predictive factor:
1. Tumor size: 6.8x4 cm.
2. Depth of invasion: Pericolorectal tissue.
3. Histologic grade: G2: Moderately differentiated.
4. Angiolymphatic invasion: Not identified.
5. Perineural invasion: Not identified.
6. Tumor deposits (discontinuous extramural extension): Not identified.
7. Surgical margins: a) Proximal Margin: Uninvolved by invasive carcinoma, distance of tumor from margin: 6.7 cm. b) Distal Margin: Uninvolved by invasive carcinoma, distance of tumor from margin: 6.5 cm. c) Radial or Mesenteric Margin: Uninvolved by invasive carcinoma, distance of tumor from margin: 3.5 cm.
8. Lymph node status: Uninvolved (0/24).
9. Extranodal involvement: Not identified.
10. Tumor regression grading S/P CCRT: No known presurgical therapy.
11. Type of polyp in which invasive carcinoma arose: Not identified.
12. Additional pathologic findings: Not identified.
13. Pathological TNM Stage: pT3N0(According to the eighth edition, 2017, American Joint Committee on Cancer Staging Guidelines for Tumors / 2017 CAP guideline).
14. TNM descriptors: Not applicable.
Gross description:
The specimen consists of a segment of colon, 11.2 cm in length, and 8.1 cm in proximal circumference, 8 cm in distal cut end circumference, and a segment of terminal ileum, 6.7 cm in length, and 4.8 cm in cut end circumference. An unremarkable appendix, 7.6x0.6 cm, is noted. A gray tan firm annular elevated and centrally ulcerative tumor, 6.8x4 cm, is noted, 6.5 cm to the distal cut end. On cross section, the tumor invaded to the mesocolon with 35 mm to the radial margin grossly, and serosal retraction is also noted. The appendix, ileocecal valve, and both cut ends are free of tumor.
Representative part for section: A) colon cut end B) ileum cut end C-H) tumor(mapping: CDE, GH) J) appendix K) tumor and ileocecal valve L-N) lymph nodes.
Note:
1. The interpretation of MMR panel and recommended studies are as below: (1) No loss of nuclear expression of MMR proteins: low probability of microsatellite instability-high (MSI-H) . (2) Loss of nuclear expression of MLH1 and PMS2: testing for methylation of the MLH1 promoter and/or mutation of BRAF is indicated (the presence of a BRAF V600E mutation and/or MLH1 methylation suggests that the tumor is sporadic and germline evaluation is probably not indicated; absence of both MLH1 methylation and of BRAF V600E mutation suggests the possibility of Lynch syndrome, and sequencing and/or large deletion/duplication testing of germline MLH1 may be indicated). (3) Loss of nuclear expression of MSH2 and MSH6: high probability of Lynch syndrome (sequencing and/or large deletion/duplication testing of germline MSH2 may be indicated, and, if negative, sequencing and/or large deletion/duplication testing of germline MSH6 may be indicated). (4) Loss of nuclear expression of MSH6 only: high probability of Lynch syndrome (sequencing and/or large deletion/duplication testing of germline MSH6 may be indicated). (5) Loss of nuclear expression of PMS2 only: high probability of Lynch syndrome (sequencing and/or large deletion/duplication testing of germline PMS2 may be indicated). (6) Please note: There are exceptions to the above IHC interpretations. These results should not be considered in isolation, and clinical correlation with genetic counseling is recommended to assess the need for germline testing.
2. This case has been peer reviewed by two doctors.
#T-59420_2 #M-81403_2 2 1105 S0000F
#T-C4520_2 #M-09183_2 0 1105 000000
#T-59200_2 #M-09183_2 0 1105 000000

病理報告　手術日期：**108/09/23**　病理號：**R0800173**

診斷醫師:陳志榮 報告日期:108/10/01
Result:
Mutation Detected.
Mutation Identified:
G13X.
Interpretation:
Mutation Detected in KRAS codon 13.
Specimen Site:
Transverse Colon.
Surgical Number:
S0833345.
Test Description:

- The KRAS Mutation Test v2 (LSR) and BRAF/NRAS Mutation Test (LSR) are allele-specific, real-time PCR test for the qualitative detection and identification of exon 2, 3, and 4 mutations in the V-Ki-ras2 Kirsten rat sarcoma viral oncogene homolog (KRAS) gene, exon 11 and 15 mutations in the proto-oncogene B-Raf (BRAF) gene and exon 2, 3, and 4 mutations in the neuroblastoma RAS viral oncogene homolog (NRAS) gene from formalin-fixed, paraffin-embedded tissue (FFPET). It is intended for life science research only and is not for use in diagnostic procedures. The mutation can be identified as KRAS G12X (G12A, G12C, G12D, G12R, G12S, G12V), KRAS G13X (G13A, G13C, G13D, G13R, G13S, G13V) , A59X (A59E, A59G, A59S, A59T), KRAS Q61X (Q61E, Q61Hc, Q61Ht, Q61K, Q61L, Q61P, Q61R), KRAS K117X (K117Nc, K117Nt) , KRAS A146X (A146P, A146T, A146V) , BRAF V600E/E2/D (BRAF V600E, V600E2, V600D), BRAF V600K , BRAF V600R , BRAF K601E , BRAF Exon11 (G466A, G466V, G469A, G469R, G469V) , NRAS G12X (G12A, G12C, G12D, G12R, G12S, G12V) , NRAS G13X (G13A, G13C, G13D, G13R, G13S, G13V) , NRAS A18T , NRAS Q61X (Q61Ht, Q61Hc, Q61K, Q61L, Q61P, Q61R) , NRAS Other Ex 3 and 4 (A59D, A59T, K117Nc, K117Nt, A146T.
  Methodology:
  The KRAS Mutation Test v2 (LSR) and BRAF/NRAS Mutation Test (LSR) are based on two major processes: (1) manual sample preparation to obtain genomic DNA from FFPET; and (2) PCR amplification and detection of target DNA using complementary primer pairs and oligonucleotide probes labeled with fluorescent dyes.
  The test is designed to detect 28 unique mutations at a percent mutation of 5% or greater, unless otherwise indicated.
  Mutation detection is achieved through PCR analysis with the cobas z 480 analyzer (Roche Diagnostics Ltd, Switzerland)... A mutant control and negative control are included in each run to confirm the validity of the run.
  Clinical Significance:
  The confirmed KRAS mutation status is critical when evaluating a candidate patient for EGFR-targeted therapy. Mutations in the KRAS gene are associated with poor prognosis and non-response to anti-EGFR therapies. KRAS mutation testing is recommended by the National Comprehensive Cancer Network (NCCN) and the Bureau Of Health Promotion, Department Of Health R.O.C before starting EGFR-targeted therapy in both metastatic colorectal cancer and advanced non-small cell lung cancer.
  Note:
  KRAS mutation test results should be interpreted in conjunction with all other available clinical and laboratory information when evaluating treatment options for cancer patients. Laboratory testing performed by the Department of Pathology of VGHTC. This test was performed with life science research reagents (LSR's). This test was developed and its performance characteristics were determined by the Department of Pathology of VGHTC. As with all LSR's, they have not been cleared or approved by the U.S. Food and Drug Administration, The FDA has determined that such clearance or approval is not necessary.
  #T-59300_2 #M-81403_2 2 1100 000000

## 其它

## 出院時情況

- 3.改門診治療

## 出院指示

| 藥名 | 劑量 | 劑量單位 | 用法 | 頻次 | 數量 | 數量單位 | 現況 | 天數 | 單位 |
|---|---|---|---|---|---|---|---|---|---|
| CefaDROxil-500 cap | 1 | CAP | PO | Q12H | 14 | CAP | TH | 7 | 天 |
| Ulstop tab 20mg | 1 | TAB | PO | Q12H | 14 | TAB | TH | 7 | 天 |
| Ultracet tab | 1 | TAB | PO | Q8H | 21 | TAB | TH | 7 | 天 |

## 出院計劃

- 門診日期
  - date on 2019/11/01

主治醫師 2222H 林俊余 / 住院醫師 2347D 許菀寧

正式摘要(電子簽章：**000000178792848)**

W52 / W52

doctorRank:B
